# Storage Stability and Consumer Acceptability of Dried Apple: Impact of Citric Acid, Potassium Sorbate and *Moringa oleifera* Leaf Extract Powder

**DOI:** 10.3390/foods11070984

**Published:** 2022-03-28

**Authors:** Washiela Arendse, Victoria Jideani

**Affiliations:** Department of Food Science and Technology, Cape Peninsula University of Technology, Bellville 7560, South Africa; washiela.arendse@gmail.com

**Keywords:** citric acid, *Moringa oleifera*, apple, browning, potassium sorbate, dried, storage, consumer acceptability, shelf life

## Abstract

The effects of a dipping solution containing 2.0% citric acid (CA) and 0.1% *Moringa oleifera* leaf extract powder (MOLEP) (CMO) and another dipping solution with CA at 2.0%, MOLEP at 0.1% and potassium sorbate (PS) at 0.2% (CMOP) on the storage stability over 3 months and consumer acceptability of dried apple slices were evaluated. Microbiological testing (osmophilic yeast, Escherichia coli and yeast and moulds) and total acidity testing were performed and physical tests, namely moisture analysis, water activity (A_w_), texture analysis and colour were performed at day 0, day 60 and day 120. Moisture increased over the shelf-life period, which affected the extensibility of the pre-treated dried sliced apples negatively. The CMO pre-treatment was effective in reducing browning and inhibiting microbial growth on the dried apple slices over the storage period. A consumer acceptability test was performed using the nine-point hedonic scale. The dried sliced apples pre-treated with the 2% CA and 0.1% MOLEP powder water solution were acceptable to consumers with regards to colour, texture and taste.

## 1. Introduction

To fully comprehend the storage life of foods, it is imperative to recognise that foodstuffs are dissimilar; they are multifaceted living systems where physicochemical, microbiological and enzymatic reactions are taking place simultaneously. The storage period, taste and texture of foods are greatly influenced by these reactions [1]. A product’s shelf life is described as a period between its production date and the point where it cannot maintain the required safety and quality criteria anymore [1,2]. The ingredients, production process and storage environment of a product plays an important role in its shelf life as well as the water activity (A_w_), temperature and the environment. All foods contain moisture, from minute quantities in dehydrated products to an excess in cold or hot drinks. There is a direct link between the speed at which food spoils and its water content, which makes it vital in the stability and shelf-life of foods [1]. The required A_w_ is obtained by the removal of moisture (dehydrating) or by adding a component (salt or sugar). Most spoilage bacteria and moulds do not proliferate at A_w_ levels below 0.91 and 0.80 [2]. Xerophytic moulds and osmophilic yeasts do not grow at water activities below 0.60 [3]. According to [1,2] water activities below 0.6 are required to avoid microbial growth. 

Oxidation is one of the main spoilage mechanisms responsible for the spoilage in food sensory characteristics. It frequently results in inferior texture, colour and flavour of the food products [4]. Physical, chemical and microbial changes are used as indicators of the quality of the foodstuffs over the storage period. The absorption of moisture by dry products leading to mushiness and sugar crystallization in dried fruit are some examples of physical changes in foods. Studies show that enzymatic, non-enzymatic browning and oxidation are the main chemical changes responsible for food spoilage and the reduction in shelf life. Microbial activity and growth in foods can lead to gas production, changes in pH, discolouration, undesirable flavours and odours [1,2]. Various methods which include consumer research, sensory trained panels and instrumental testing are used to assess whether sensory attributes of foods and beverages are altered over time [5]. Instruments can be used to examine the texture and sensory changes such as rancidity or pH shifts in the place of consumer research [2]. According to [6,7], when drying is used as a preservation method, sulphur dioxide is normally used as a preservative to maintain the quality of foodstuffs. However, the safety of food processed with sulphites has been questioned because of its harmful effects on health, particularly in asthmatic individuals [8,9]. 

Food producers use sensory studies as quality criteria, to determine product attributes and shelf life and to measure the acceptability of a product when developing new products. The sensory characteristics usually evaluated are appearance, odour, taste and texture [4]. Sensory studies as practised presently are still in their infancy. The beginning of the 1940s saw the growth of hedonic food acceptability methods; the use of the nine-point hedonic scale developed by the US Army Corps of Engineers became standard practice for several US food companies. Sensory analysis, however, has been practised since the 1800s with the advancement of research into human psychology to calculate and foresee an individual’s reactions to outside stimuli [10,11]. Sensory evaluation is defined as a scientific approach whereby the reactions of individuals to foodstuff are awakened, calculated, translated and evaluated by their sense of sight, taste, touch and hearing [5,12]. Its fundamental goal, whether using trained panels for quality purposes or acceptance using consumers, is to gain insight into a human’s view on how they experience the consumption of food using their senses (sight, taste, touch and hearing). In addition to taste and odour, humans also experience trigeminal sensations or pain responses produced by chemical irritants in products such as capsaicin in chillies, menthol, alcohol and ginger. 

Together with taste, odour and trigeminal sensations are referred to as flavour. In the food industry, sensory statistics or information are used to determine quality standards, consumer acceptability, for product development and shelf-life studies. Sensory science allows food manufacturers to consistently produce and develop good quality products [4,5,10,12]. Sensory studies provide data on which sound decisions can be made [5]. 

A sensory evaluation has two major classifications namely, analytical and hedonic or affective testing. Analytical assessments are used to assess if an overall difference exists between two or more products, where no characteristic can be recognised as being affected. The most renowned analytical sensory test is the difference or discrimination test such as difference from control and paired comparison and triangle tests [5,11,12]. The different tests which are used most extensively are the triangle and duo-trio tests [11,12]. Depending on the objective of the sensory evaluation, the number of panelists proposed for analytical discrimination testing is 25–50 [11]. Quantitative descriptive analysis (QDA) tests are some of the most regularly used profiling procedures and use trained panelists to carry out these methods [5,12]. For the QDA based procedures, a minimum of eight screened, selected and trained assessors are recommended [5]. 

Hedonic tests have two classifications namely, preference and acceptability tests. As the names indicate, these tests merely measure the degree of liking and preference. If the objective of the research is to set one product against another, then the preference test should be used. Two or more samples may be used; consumers are asked to rank their preference if a sample size greater than two are used [5,11]. The weakness of this assessment is that the degree of liking is not established [11]. When the objective is to assess how much a product is liked by a consumer, then the acceptance test should be used to indicate the degrees of like or dislike, of which the nine-point hedonic scale is generally used. It shows an acceptance on a nine-point numerical scale labelled from “dislike extremely” to “like extremely”. At least fifty consumers are suggested for acceptance testing. Consumers used for this test should be untrained and regular end-users of the product tested [5,11]. 

A study by [13] showed that a pre-treatment with 2.0% CA, 0.1% MOLEP and a dehydrating for 7 h at 70 °C minimized the browning of the dried sliced apples. Even though, according to the study by [13], citric acid (CA) was more effective than potassium sorbate (PS), it was thought it may assist to increase the storage period of the dried apple slices. However, nothing is known about the impact of the dipping solution with potassium sorbate on the shelf-life and consumer acceptability of dried sliced apples. Therefore, this research aimed to establish the impact of the dipping solution (2.0% CA and 0.1% MOLEP and dipping solution with 0.2% PS) on microbiological safety as well as the physicochemical properties of the dried apple slices during storage and to establish consumer acceptability using the nine-point hedonic scale. 

## 2. Materials and Methods

### 2.1. Source of Materials and Equipment

The apples (Granny Smith) were bought from a store in South Africa (Cape Town). The apples were stored at 4 °C + 1 °C until required for the trial [14,15,16,17]. *Moringa oleifera* leaf extract powder was bought in South Africa from Dohler Pty (Ltd.) (Paarl), CA from Savanna Fine Chemicals (Pty) Ltd., Cape Town, South Africa) and PS from Bragan Chemicals CC, South Africa (Johannesburg)). A cabinet dehydrator (Excalibur, model no: EXC 10) was used to dried the sliced apples. The dehydrator was composed of a fan (horizontal airflow), ten trays, a heating element and a thermostat situated at the rear of the dryer. Cold air was drawn in, warmed and circulated uniformly across every tray [18].

### 2.2. Preparation and Drying Procedure of the Apples

Before drying the apples, they were physically washed and sanitised in a 200 ppm sodium chlorite solution [14]. The apples were then peeled, de-cored and sliced to approximately 4 mm in thickness [14,16,19,20]. The prepared sliced apples were dipped in a water solution of 2.0% CA and 0.1% MOLEP (CMO) and another solution of 2.0% CA, 0.1% MOLEP and 0.2% potassium sorbate (CMOP). The apple slices were submerged for 5 minutes in the CMO and CMOP solutions [17]. After the pre-treatment, the excess solution was softly patted off with tissue paper [9,14]. The untreated (control) and pre-treated apple slices were positioned evenly and as a single layer on the trays and dried for 7 h at 70 °C. 

### 2.3. Shelf Life Stability Assessment of the Dried Apple Slices

The untreated and pre-treated dried apple slices were packed in pouches (polyester, foil and low-density polyethylene), heat-sealed and stored under ambient/room temperature [20,21]. The dried apple slices were stored for 120 days [22,23,24]. The dried apple slices were tested for colour (L*, a*, b*), water activity, moisture, yeasts and moulds, osmophilic yeasts, Escherichia coli, total acidity and texture on the day of production (day 0), day 60 and day 120. 

### 2.4. Colour Measurement

The dehydrated sliced apple’s (pre-treated and control) surface colour was measured with a spectrophotometer (Spectrocolorimeter Datacolor 600). This equipment determines the trichromatic coordinates CIELAB (L* a* b*) using a spectrophotometer with measurement geometry d/8 (diffuse illumination, directional observation at 8° + 2°) and D65 illuminant. A black trap and a white tile were used to calibrate the equipment before analyses. The dried sliced apple was positioned completely flat on the instrument’s reading surface. The analysis was performed on ten randomly selected dried sliced apple pieces. The colour was documented as L*, a*, and b* values. L* (lightness) ranges from 0–100, 0 being black (no light) and 100 white (upper limit). The a* is green (negative) to red (positive). The b* is blue (negative) to yellow (positive) [25,26]. The whiteness index (WI) was determined using the L*, a* and b* values and Equation (1) [27,28]. The L*, a* and b* values and Equation (2) were used to determine the browning index (BI) [29].

(1) $$\mathrm{WI}=100-\sqrt{{\left(100-L^{*}\right)}^{2}+a^{*}{}^{2}+b^{*}{}^{2}}$$

where: L* (lightness), a* (redness) and b* (yellowness) are colour measurements of the dried apple slices.

(2) $$\mathrm{BI}=\frac{\left[100\;\left(x-0.31\right)\right]}{0.172}$$

where

$$x=\frac{\left(a^{*}+1.75L^{*}\right)}{5.646L^{*}+-3.012b^{*}}$$

### 2.5. Total Acidity Determination

The total acidity of the dried apple slices was determined using an automatic titrator (METROHM). The analysis was performed on 10 randomly selected dried apple slice pieces. The apple slices were ground, and 4 g was weighed and dissolved in boiled distilled water and titrated with NaOH 0.1 N at room temperature using an automatic titrator. Total acidity was recorded as g citric acid monohydrate/100 g.

### 2.6. Water Activity (A_w_) Determination

The ISO 21807:200 method for foodstuffs which measure dew point was used to determine the A_w_ in the dried apple slices at 25 °C. The instrument was calibrated using salt solution standards with known water activity. A representative sample was placed in a cup placed in the instrument (Aqualab) and sealed. The final aw reading was displayed on the screen of the instrument. The analysis was based on the correlation between the air dew point in equilibrium with the sample and its free water content. 

### 2.7. Moisture Determination

Moisture was determined using the Karl Fischer titration method. The analysis was performed on 10 randomly selected dried apple slice pieces which were ground, and a 4 g sub-sample was weighed and dissolved in 40 g of methanol formamide mixture and titrated with Karl Fischer reagent at room temperature. The final titration mark was determined electronically. 

### 2.8. Microbiological Analysis

The dried apple slices were tested for yeast and mould, osmophilic yeast and *Escherichia coli* [30]. A 10 g representative sample was added to 90 g of buffered peptone water (10-1 dilution). One gram of the inoculum was plated onto the respective agars stated in Table 1. Thereafter, the plates were incubated for the periods and temperatures as specified for the respective microorganisms as indicated in Table 1. Results were reported as cfu/g (colony-forming unit per gramme). 

### 2.9. Texture Analysis

Texture analysis was done with a texture analyser (TA. XT plus Texture analyser-Stable Micro System). The analysis was performed on 10 randomly selected dried apple slice pieces. A metallic cylinder probe 4 mm in diameter, a force of 5 g and a test speed of 1 mm/s was used to test the extensibility of the dried apple slices. The extensibility results were reported in millimeters.

### 2.10. Consumer Acceptability Testing

**Figure 1:**
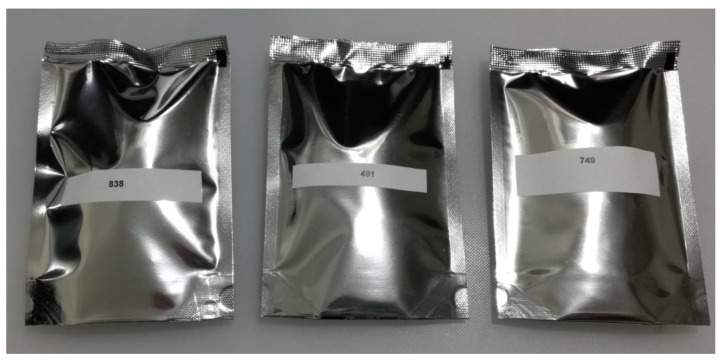
Randomly coded aluminium foil pouches containing the dried apple slices.

The dried apple slices pre-treated with CMO and CMOP and the control were weighed (20 g/sample) and packed in aluminum foil pouches and heat sealed. The samples were labelled with random number codes. An acceptability test was done using the nine-point hedonic scale. Fifty-six consumers participated in the study. The participant’s pool consisted of Cape Peninsula University of Technology (CPUT) staff and students as well as external consumers. The sensory test was conducted in CPUT’s sensory facility. The sensory test room was regulated with an air-conditioner, run at 18 °C, with white light. The consumers each received on a tray three (control, CMO and CMOP) 20 g randomly coded dried apple sliced samples, packed in aluminum foil pouches as indicated in Figure 1. A cup with water, to clean the palate between tastings was provided. The consumers were asked to complete the questionnaire and to indicate the acceptability of the samples in terms of colour, texture and taste on the nine-point hedonic scale, where one indicates “dislike extremely” and nine indicates “like extremely”. 

### 2.11. Statistical Analysis

Significant differences between treatments were evaluated by multi variate analysis of variance (MANOVA). Duncan’s multiple range test was used to separate the means where differences existed at *p* < 0.05. Sensory ratings were expressed as mean ± standard deviation. The data were tested for normality using the Shapiro–Wilk test and homogeneity of variance by the Levene’s test in IBM SPSS version 26. Where normality assumption was violated, sensory differences between samples was estimated using the non-parametric analysis of variance equivalent, the Kruskal–Wallis test. Results were analysed using DesignExpert version 12 software.

## 3. Results and Discussions

### 3.1. Impact of the Pre-Treatments on the Colour and Storage Time of the Dried Sliced Apples

Table 2 shows the impact of the pre-treatment on the colour of the dehydrated apple slices. The F-value for lightness was indicated as F (2,81) = 30.7; *p* = 0.00, redness F (2,81) = 25.7; *p* = 0.00 and yellowness F (2,81) = 132.6; *p* = 0.00. The lightness of the dehydrated sliced apples pre-treated with CMO (2.0% CA and 0.1% MOLEP), L* = 85.6 was significantly (*p* = 0.000) higher than the CMOP (2.0% CA, 0.1% MOLEP and 0.2% PS), L* = 80.8 and the control (L* = 79.6) at day 0. Similarly, the lightness of the CMO (L* = 80.1) dried apple slices was significantly (*p* = 0.022) higher than CMOP (L* = 78.4) and the control (L* = 75.0) at day 120. The redness of the CMO (a* = 1.5) dried apple slices was significantly (*p* = 0.000) lower than the CMOP (a* = 4.0) and the control (a* = 4.8) at day 0.

Likewise, the redness of the CMO (a* = 3.9) dried apple slices was significantly (*p* = 0.018) lower than the CMOP (a* = 6.2) and the control (a* = 7.0) at day 120. Yellowness of the CMO (b* = 17.9) dried apple slices was significantly (*p* = 0.000) lower than the CMOP (b* = 20.5) and the control (b = 33.3) at day 0. At day 120, yellowness of the CMO (b* = 28.4) and the CMOP (b* = 25.1) dehydrated sliced apples were significantly (*p* = 0.000) less than the control (b = 33.8). 

Thus, CMO dried apple slices was significantly lighter, and less red and yellow than the control over the storage time. Also, the CMO dried apple slices was significantly lighter and less red over the storage period than the CMOP. 

Table 3 details the effect of storage time on the lightness (L*), redness (a*) and yellowness (b*) of the pre-treated and untreated dried apple slices. The lightness of the dried apple slices pre-treated with CMO was at day 0 (L* = 85.6) and day 60 (L* = 85.1) significantly (*p* = 0.000) higher than at day 120 (L* = 80.1). Similarly, the redness of the dried apple slices pre-treated with CMO at day 0 (a* = 1.5) and day 60 (a* = 0.9) was significantly (*p* = 0.000) lower than at day 120 (a* = 3.9). It was observed that the yellowness of the CMO sliced apples at storage day 0 (b* = 17.9), day 60 (b* = 21.7) and day 120 (b* = 28.4) was significantly (*p* = 0.00) different. The yellowness of the CMO dried apple slices at day 0 was significantly lower than at day 120. Therefore, the CMO dried apple slices were significantly lighter and less red at day 0 and day 60 of storage than at the end (day 120), whereas the yellowness significantly increased over the storage time. 

**Figure 2:**
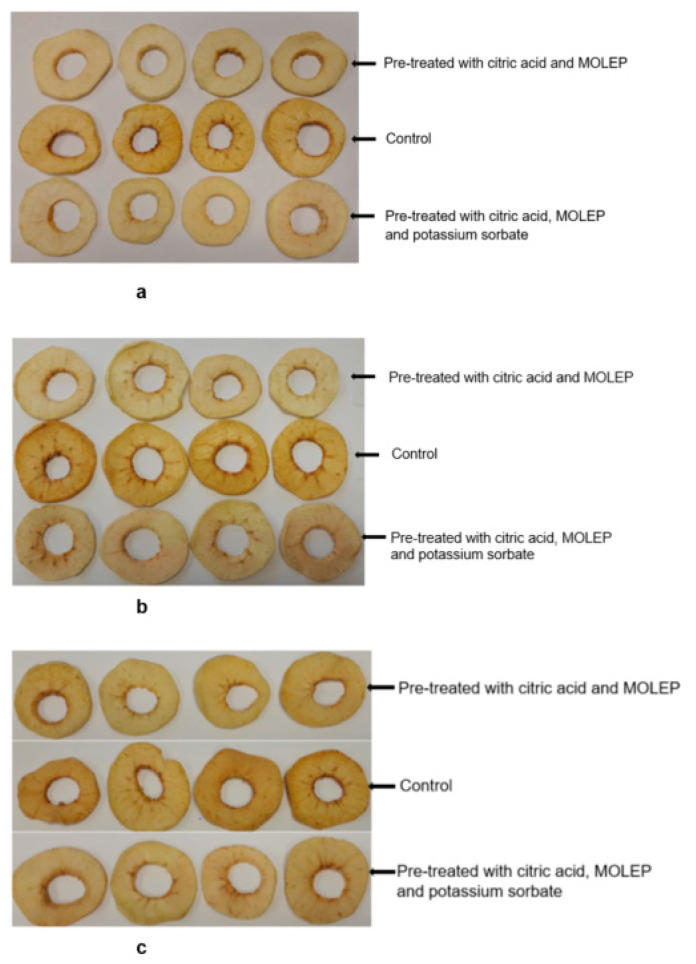
Image of untreated and pre-treated dried apple slices at storage time (**a**) 0 days; (**b**) 60 days and (**c**) 120 days.

The lightness of the dried apple slices pre-treated with CMOP at day 0 (L* = 80.8), day 60 (L* = 80.1) and day 120 (L* = 78.4) was not significantly different (*p* = 0.139). However, the redness of the CMOP dried apple slices was significantly higher (*p* = 0.007) at day 120 (a* = 6.2) than at 60 (a* = 3.9) and day 0 (a* = 4.0). Similarly, the yellowness of the CMOP apple slices was significantly (*p* = 0.000) higher at day 120 (b* = 25.1) than at day 60 (b* = 21.5) and day 0 (b* = 20.5). Therefore, the CMOP dried apple slices were more red and yellow at the end of the storage time while no significant change in the lightness over the storage period was observed. Figure 2 illustrates the untreated and pre-treated dried apple slices at storage days 0, 60 and 120. 

### 3.2. Impact of the Pre-Treatments on the Browning Index (BI), Whitening Index (WI) and Storage Time of the Dried Sliced Apples

The effect of the pre-treatment on the browning and whitening index of the dried apple slices is shown in Table 4. The BI of the dried sliced apples pre-treated with 2.0% CA and 0.1% MOLEP (CMO) was significantly (*p* = 0.000) different over the storage time. The BI of the CMO dried apple slices was 23.9 at storage day 0, 29.4 at day 60 and 46.4 at day 120. However, BI for the CMO dried apple slices was significantly (*p* = 0.000) lower than the BI of CMOP (2.0% CA, 0.1% MOLEP and 0.2% PS) which was 32.0 at day 0 and 34.0 at day 60 while at day 120 (BI = 43.6) there was no significant difference between CMO and CMOP. The CMO dehydrated sliced apples BI were significantly (*p* = 0.000) less than the control, which was 57.5 at day 0, 61.0 at day 60 and 66.1 at day 120 (*p* = 0.001). Even though the browning index of the CMO and CMOP dried apple slices increased overtime it was significantly less brown than the control at the beginning (*p* = 0.000) and end (*p* = 0.001) of the storage time. 

The whitening index (WI) of the CMO dehydrated sliced apples was significantly (*p* = 0.000) different over the storage period. The WI for CMO was 76.9 at day 0, 73.6 at day 60 and 65.0 at day 120. Though the CMO dried apple slices WI was significantly higher than CMOP at day 0 and 60 which were 71.6 at day 0 and 70.4 at day 60 there was no significant difference observed at day 120 (WI = 66.2). The WI of the CMO dried apple slices was significantly higher than the control which were 60.6 at day 0, 58.6 at day 60 and 57.2 at day 120. Thus, the WI of the CMO and CMOP dried apple slices decreased over the storage time but was more evident at day 120. Although the CMO and CMOP dried apple slices whitening index decreased over time it was significantly whiter than the control at the start (*p* = 0.000) and the end (*p* = 0.001) of the storage time. 

The effect of storage time on the BI and WI of the dried apple slices is indicated in Table 5. The BI of the dried apple slices pre-treated with CMO at day 0 (BI = 23.9) and day 60 (BI = 29.4) was significantly (*p* = 0.000) lower than day 120 (BI = 46.4 ± 11.0). Similarly, the BI of CMOP at day 0 (BI = 32.0) and day 60 (BI = 34.0) was significantly (*p* = 0.000) lower than day 120 (BI = 43.6 ± 7.5). The BI of the control was not significantly (*p* = 0.421) different over the storage time. The dried apple slices pre-treated with CMO at day 0 (23.9) and 60 (29.4) BI was lower than CMOP (day 0 = 32.0 and day 60 = 34.0). However, at day 120 the dried apple slices pre-treated with CMO (BI = 46.4) was higher than CMOP (BI = 43.6) though not significantly (*p* = 0.001). 

It was observed that the BI of the CMO dehydrated sliced apples was less than that of the control at day 0 (57.5), day 60 (61.0) and 120 (66.1). Thus, dried apple sliced pre-treated with the 2% citric acid and 0.1% Moringa oleifera (CMO) were less brown at days 0, 60 and 120 compared to the control over the same period. Furthermore, CMO dried apple slices were less brown than the CMOP (2.0% CA, 0.1% MOLEP and 0.2% PS) at days 0 and 60 with no significant difference noted at day 120. The whitening index (WI) of the dried apple slices pre-treated with CMO (2.0% CA and 0.1% MOLEP) at day 0 (WI = 76.9) and day 60 (WI = 73.6) was significantly (*p* = 0.000) higher than day 120 (WI = 65.0). Likewise, the WI of CMOP at day 0 (WI = 71.6) and day 60 (WI = 70.4) was significantly (*p* = 0.002) higher than day 120 (WI = 66.2), whereas that of the control was not significantly (*p* = 0.371) different over the storage time. An observation was made that the WI of the dehydrated sliced apples pre-treated with CMO at days 0 and 60 was higher than CMOP. However, the WI of the dried apple slices at day 120 pre-treated with CMOP was higher, though not significantly (*p* = 0.001). Thus, dried apple sliced pre-treated with the 2.0% CA and 0.1% MOLEP (CMO) were whiter at days 0, 60 and 120 compared to the control over the same storage period. Additionally, CMO dried apple slices were whiter than the CMOP (2.0% CA, 0.1% MOLEP and 0.2% PS) at days 0 and 60 with no significant difference at day 120 observed. The CMO dried apple slices showed a significantly higher value in lightness, and a lower value in redness and yellowness over the storage time compared to the control. The browning index (BI) of the CMO dried apple slices increased significantly while that of the whitening index (WI) declined significantly over the storage period. However, the BI of the CMO dehydrated sliced apples were significantly lower and the WI significantly higher than the control. 

The favourable results of CA on the colour (a* and b*) of the dehydrated sliced apples can be attributed to its ability to lower pH and chelate metals [31,32]. The enzyme polyphenol oxidase (PPO) is dependent on copper to operate, according to [33]. The author [31] reported that the bond of the copper at the active site of PPO weakens at pH values < 4. Since the pH of the pre-treatments were <4 (CMO–2.16 and CMOP–2.41), copper ions can consequently be separated from the active site of polyphenol oxidase and browning can thus be averted [27,34,35]. 

The study by [36] reported that Moringa oleifera (MO) leaves are a great source of organic antioxidants and are able to extend the shelf life of foodstuffs that has fat as an ingredient. Flavonoids are the key phytochemical constituents in the MO leaves [37,38]. Ref. [39] reported that MO leaves are an exceptional source of phenolic components and an effectual antioxidant. According to [39], 0.1% MOLEP was enough to stop the oxidation of fat in goat meat patties that were kept in a refrigerator over time. This agrees with the research by [36] that reported MO leaves at reduced quantities showed moderately better antioxidant activity over time. The phytochemicals, specifically the flavonoids present in the MO leaves, might thus be the reason for the effective antioxidant activity of the MOLEP. 

The CMOP pre-treatment indicated better lightness and yellowness compared to the control for the storage period; however, CMO dried apple slices showed significantly higher lightness and lower redness when compared to the CMOP dried apple slices. The difference in yellowness between the CMO and CMOP dried apple slices was not significant during the storage time. Initially, the BI (day 0) and WI (day 0 and 60) of the CMO dried sliced apples was superior to that of CMOP; however, at the end of the storage, the difference was not noticeable. According to [40], sorbic acid is susceptible to oxidation and degradation by first-order reaction kinetics in aqueous solutions. Furthermore, the breakdown of sorbates occurs with prolonged heating, since the apple slices were dried for 7 h at 70 °C (prolonged heat treatment), which might be another reason why CMOP pre-treatment was not as effective as the CMO pre-treatment in reducing the discolouration (L*, a*) of the dried sliced apples during storage. 

### 3.3. Impact of the Pre-Treatments on the Microbial Count of the Dried Sliced Apples during Storage

The microbial count for osmophilic yeasts, yeasts and moulds and *E. coli* at storage days 0, 60 and 120 are presented in Table 6. The osmophilic yeasts count for the control, CMO and CMOP was <100 cfu/g and the *E. coli* count for the control, CMO (2.0% citric acid and 0.1% MOLEP) and CMOP (2.0% citric acid, 0.1% MOLEP and 0.2% potassium sorbate) dried apple slice samples were <10 cfu/g over the storage period. The initial (day 0) yeasts count for the CMO (<100 cfu/g) dried apple slices showed a one log reduction and CMOP pre-treated dried apple slices showed a 2 log (<10 cfu/g) reduction compared to the control (<1000 cfu/g). The mould count of the CMO (140 cfu/g) dried apple slices at storage day 0 showed a 2 log reduction and the CMOP (40 cfu/g) pre-treated sample showed an even further reduction when compared to the control (14,000 cfu/g) at the same storage time. At 120 days, the yeasts and moulds count for all three samples (control, CMO and CMOP) were <10 cfu/g. Yeasts and moulds are the leading cause of microbial spoilage in dried fruit [8,41]. This research showed that the CMO and CMOP pre-treatment reduced the initial (day 0) yeasts and moulds count of the dehydrated sliced apples. The impact of the CMO and CMOP pre-treatments on the yeast and moulds count of the dried apple slices showed better results than the untreated (control); however, the effect of the CMOP pre-treatment on the yeasts and moulds count was more effective than that of the CMO pre-treatment. 

Microorganisms react to low pH stress by stopping a harmful decrease in intercellular acid levels below the limit required for viability. One such strategy microbes practise to avert a serious fall in the pH level is to employ enzyme-catalysed reactions that use protons [42]. Thus, as a result of the low pH (<4) of the CMO and CMOP water solution used as a pre-treatment, the enzymes needed to maintain this critical activity of the microbes are inactivated by the formation of covalent bonds between the weak acids and the enzymes, causing the metabolic actions of the microbes to stop [8].

### 3.4. Impact of the Pre-Treatments on the Total Acidity, Water Activity, and Moisture of the Dried Sliced Apples during Storage

The summary of the mean values for the impact on the total acidity, A_w_ and the moisture of the dehydrated sliced apples over time is presented in Table 7. Total acidity (TA) of the dehydrated sliced apples ranged for the control from 2.1–1.5, for CMO from 3.8–2.9 and CMOP from 3.0–4.1 over the storage period. The dried apple slices initial TA of the CMO (3.8) and CMOP (3.0) was higher than the control (2.1) with CMO showing the highest result. The total acidity of the control decreased by 29% and CMO by 9.6%, whereas the CMOP dried apple slices increased by 39.1% over the storage period. The A_w_ of the dehydrated sliced apples ranged for CMO from 0.3–0.5, for CMOP from 0.3–0.4, and the control remained unchanged at 0.4 over the storage period. 

The initial (day 0) water activity of the CMO (A_w_ = 0.3) and CMOP (A_w_ = 0.3) dried apple slices was lower compared to the control (A_w_ = 0.4). A substantial increase of 32.4% in the A_w_ of the CMO dried apple slices was noted while that of CMOP increased by 9.1% and that of the control decreased by 2.7% over the storage period. The moisture of the dried apple slices for the control over the storage period ranged from 7.3–7.8%, for CMO, from 6.0–10.4% and for CMOP, from 6.1–7.7%. A decrease in the initial moisture of the CMO (6.0 %) and CMOP (6.1%) dried apple slices was observed when compared to the control (7.3%) with CMO showing the highest increase over time. The moisture of the CMO dried apple slices showed a considerable increase of 73.6% while CMOP increased by 26.9% and the control increased by 6.5% over the storage period. 

According to [16], apples pre-treated with citric acid have a higher moisture diffusivity rate than untreated samples. Since all the dehydrated sliced apples were dried at the same temperature and time, the initial lower water activity (aw) and moisture observed in the apple slices pre-treated with CMO and CMOP can be due to the citric acid present in the pre-treatment solutions. According to [43,44], no spoilage bacterial growth occurs at water activity levels < 0.95 and generally no proliferation of yeast and mould takes place at water activity levels < 0.61. The A_w_ for the CMO sample ranged from 0.3–0.45 and for the CMOP sample from 0.33–0.36, which is well within the water activity levels not conducive to bacterial, yeast and mould growth. 

As expected, the total acidity of the dried apple slices pre-treated with 2% citric acid, 0.1% *Moringa oleifera* (CMO) and 2% citric acid, 0.1% *Moringa oleifera* and 0.2% potassium sorbate (CMOP) increased; this can be ascribed to the presence of CA and PS in the pre-treatment solutions. According to [45], the acidity of fruit plays a vital part when assessing the flavour of the fruit. It was observed in this study that the total acidity of the CMO (9.6%) dried apple slices changed the least over the storage period, whereas the CMOP (39.1%) showed the highest increase. This suggests that the CMO dried apple slices might have the least change in its flavour profile. 

The moisture of the control, CMO and CMOP samples increased over the storage period, which may be a result of samples absorbing moisture from the environment. Even though the moisture of the CMO dried apple slices increased considerably over the storage period, it was still within the moisture limit of <27% as prescribed by regulation 653, relating to the quality, packing and marketing of dried fruit intended for sale in the Republic of South Africa. 

### 3.5. Effect of the Pre-Treatments on the Extensibility and Storage Time of the Dried Sliced Apples

The effect of the pre-treatment on the extensibility of the dried sliced apples is presented in Table 8. The extensibility of the CMO (4.1 mm), CMOP (4.7 mm) and control (4.7 mm) was not significantly (*p* = 0.099) different at day 0. It was noted that the CMO (5.1 mm), CMOP (6.3 mm) and control (4.5 mm) dried apple slices extensibility was significantly (*p* = 0.000) different at day 60 with the CMO and CMOP showing higher results than the control. However, the extensibility of the CMO dried apple slices were significantly lower than CMOP at the same time. However, this changed at day 120 with the CMO (8.0 mm) dehydrated sliced apples showing a significantly (*p* = 0.000) higher extensibility than the CMOP (7.0 mm) and the control (6.9 mm). Thus, the dried apples pre-treated with 2.0% citric acid and 0.1% MOLEP (CMO) and 2.0% citric acid, 0.1% MOLEP and 0.2% potassium sorbate (CMOP) became less crispy and chewier during the storage period.

The impact of storage period on the extensibility of the dehydrated sliced apples is indicated in Table 9. The overall effect over time on the extensibility of the dehydrated sliced apples was significant (*p* = 0.000). It was noted that the CMO dehydrated sliced apples’ extensibility significantly (*p* = 0.000) increased over the storage time (day 0 = 4.1, day 60 = 5.1and day 120 = 8.0). Likewise, the CMOP dried apple slices also significantly (*p* = 0.000) increased over the storage period (day 0 = 4.7, day 60 = 6.3 and day 120 = 7.0). Therefore, the CMO and CMOP dried apple slices are less crunchy and leatherier over time.

Research by [46] stated that moisture content affects the texture of dried fruit during storage. A substantial increase in moisture of the dried apple slices pre-treated with CMO (moisture increase = 73.6%) and CMOP (moisture increase = 26.9%) was noticed over the storage period. This increased moisture can cause a reduction in the crispness of the dried apple slices and can thus be the reason for the increase in the extensibility (texture) over the storage period [25,47]. A report by [16] indicated that pre-treatments with citric acid reduces the drying time of foods and according to [48], the increased firmness/hardness is an effect of the higher drying temperature which accelerates the removal of moisture from the tissue. An increase in extensibility was observed in the dehydrated apple slices pre-treated with CMO and CMOP. The increased extensibility of the pre-treated samples (CMO, CMOP) can thus also be linked to the presence of citric acid and the method used to dry the apple slices. 

### 3.6. Consumer Acceptability

**Figure 3:**
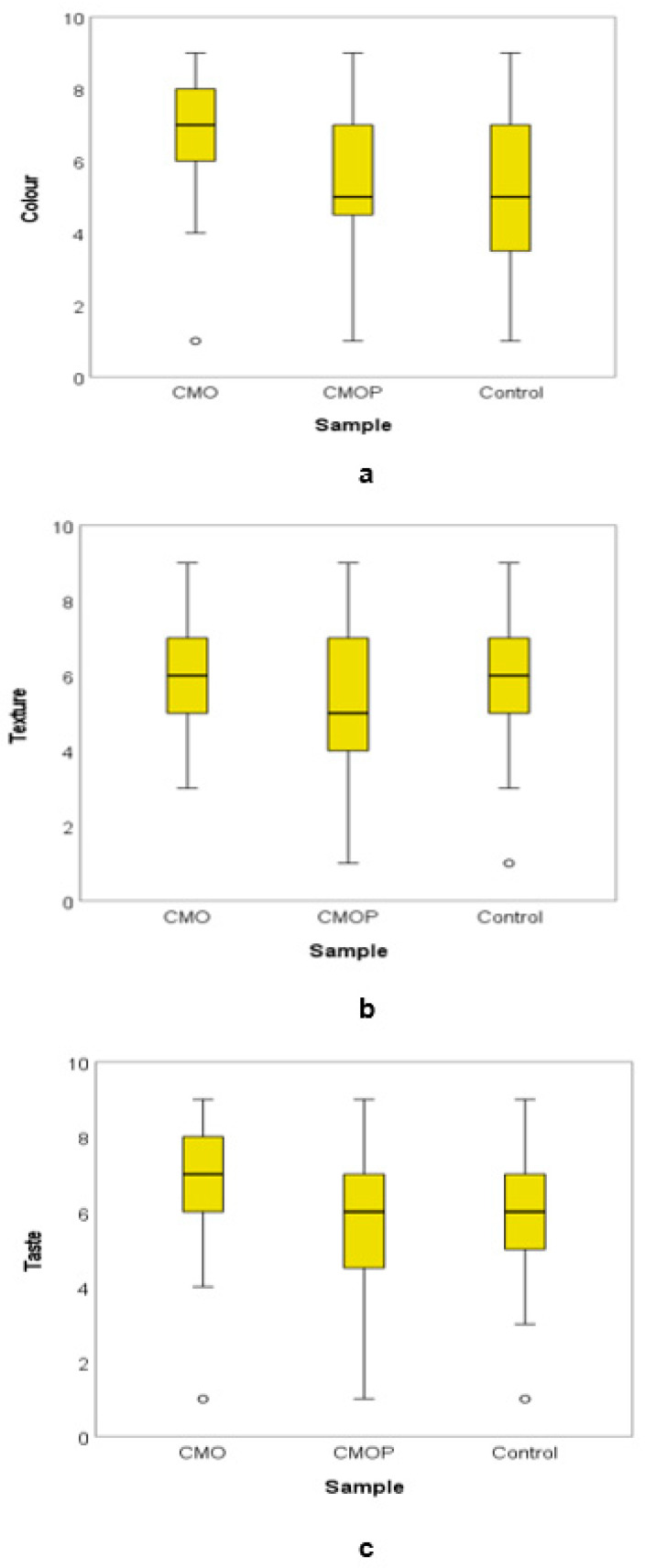
Mean value scores of acceptability: (**a**) colour, (**b**) texture and (**c**) taste scores for CMO (Citric acid + *Moringa oleifera* leaf extract powder), CMOP (Citric acid + *Moringa oleifera* leaf extract powder + potassium sorbate) and control dried sliced apples.

Fifty-six consumers completed the questionnaire, of which 51.8% were female, 48.2% were male, 16.1% were in the age group 18–25, 8.9% were ages 26–30, 10.7% were ages 31–35 and 64.3% were >36 years of age. All the panelists indicated their consumption frequency of dried apples as once a month. The mean values of the consumer’s acceptability scores for colour, texture and taste are shown in Figure 3. The mean of colour scores for CMO was 6.8, whereas for CMOP it was 5.3 and for the control, 5.1. The texture scores indicate mean values of 6.1 for CMO, 5.2 for CMOP and 5.6 for the control. The mean value for taste acceptability was 6.5 for CMO, while CMOP was 5.9 and the control was 6.1. The colour acceptability of the CMO dried apple slices was significantly (*p* = 0.00) higher than the colour of CMOP and the control. Similarly, for texture, the CMO acceptability was significantly (*p* = 0.029) higher than CMOP and the control dried apple slices. 

**Figure 4:**
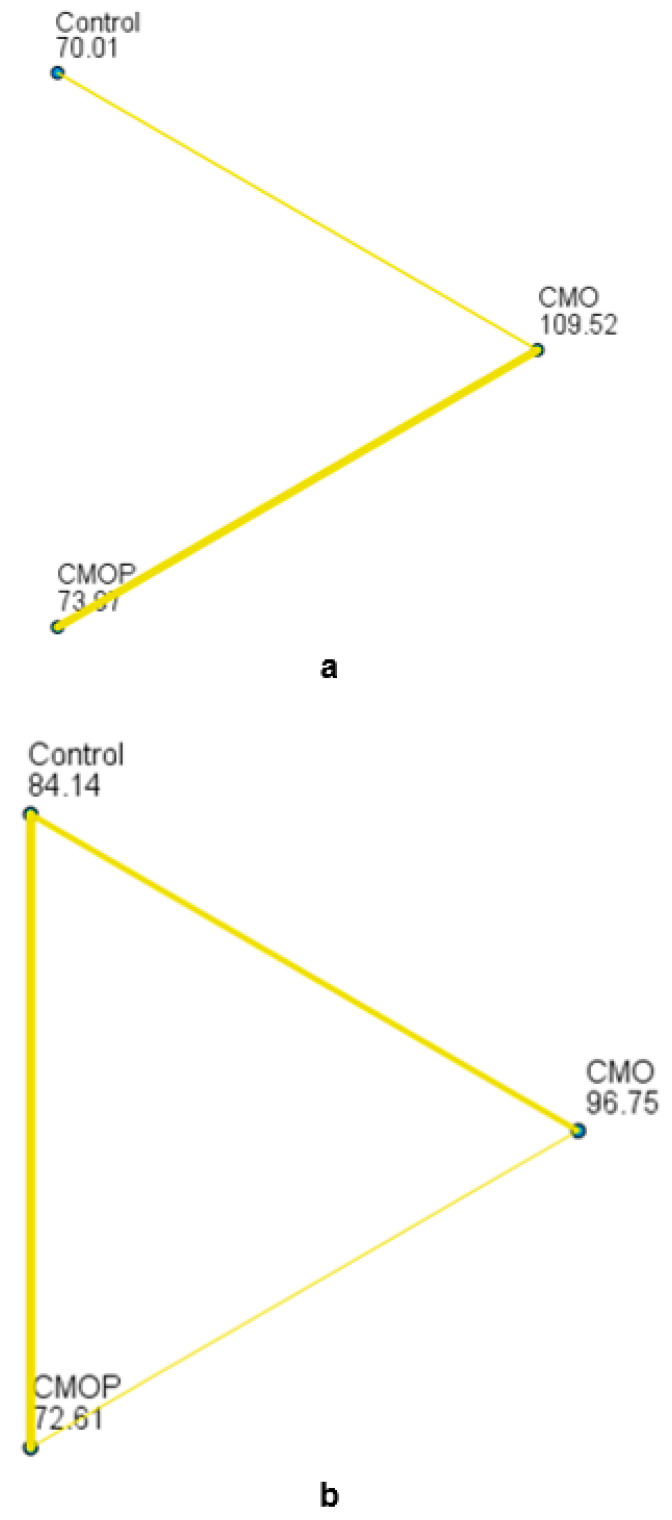
Paired comparison of the samples. Each node shows the sample average rank for (**a**) colour and (**b**) texture of the CMO (Citric acid + *Moringa oleifera* leaf powder), CMOP (Citric acid + *Moringa oleifera* leaf powder + potassium sorbate) and control dried sliced apples.

The study showed that the pre-treatment did not impact the taste (*p* = 0.161) of the dried apple slices negatively. The pairwise comparison of the dehydrated sliced apples indicated no significant difference (*p* = 0.663) in colour for CMOP compared to the control; however, colours of the control and CMO were significantly different (*p* = 0.000). The data analysis showed that the acceptability of the dried apple slices colour between the CMO and CMOP was significantly (*p* = 0.000) different. A statistically significant (*p* = 0.000) difference in colour between the pre-treated (CMO, CMOP) and untreated (control) dried apple slices were observed with the mean colour ranking score for CMO being the highest at 109.52, followed by CMOP at 73.97 and the control at 70.01 (Figure 4a). 

The pairwise comparison results for texture indicated no significant (*p* = 0.203) difference for CMOP compared to the control as well as no significant (*p* = 0.164) difference between the CMO and the control sample. A significant (*p* = 0.008)) difference was noted between the CMO and CMOP dried apple slices for texture. The average ranking results for texture between the pre-treated (CMO and CMOP) and untreated (control) dried apple slices were also significant (*p* = 0.029). The CMO dried apple slices are ranked highest for texture (96.75), followed by CMOP at 84.14, and the control at 72.61 (Figure 4b). The rankings indicated that the panelist perceived the colour and texture of the CMO dried apple slices as better than those of the CMOP and control. Multiple comparisons for taste were not performed because the overall test did not show significant (*p* = 0.161) differences across the control, CMO and CMOP dehydrated sliced apples. The colour of CMO dehydrated sliced apples was liked moderately (6.8) and texture liked slightly (6.1), whereas the colour of the control was neither liked nor disliked (5.1), though the texture was liked slightly (5.6) by the consumers. The colour (5.3) and the texture (5.2) of the CMOP dried apple slices were neither liked nor disliked. The taste of the CMO dried apple slices was liked moderately (6.5), while the taste of both CMOP (5.9) and control (6.1 + 1.8) was liked slightly by the consumers. The CMO dried apple slices were ranked the highest for colour (109.52) and texture (96.75). The CMOP dried apple slices ranked second highest for colour (73.97) whereas the control (84.14) was ranked the second highest for texture. The colour (*p* = 0.000 < 0.05) and texture (*p* = 0.029) across samples (CMO, CMOP and control) were significantly different, whereas for taste it was not significantly different (*p* = 0.161). 

Therefore, the CMO dried apple slices was liked more than the CMOP and control. Hot air drying of plant material often results in a decline in the quality of dried foodstuff, presenting unwanted differences in sensorial properties such as appearance, texture and taste [49]. The consequence of dehydration using hot air inevitably leads to the worsening of surface browning and affects the sensorial properties of dried apple products. According to [50,51], browning also influences consumer liking, buying behaviour and consumption of the products. Consequently, dehydration of fruit might produce unacceptable changes in physicochemical attributes such as colour changes, and differences in form and volume that could cause reduced consumer acceptance and value perception [51]. 

**Figure 5:**
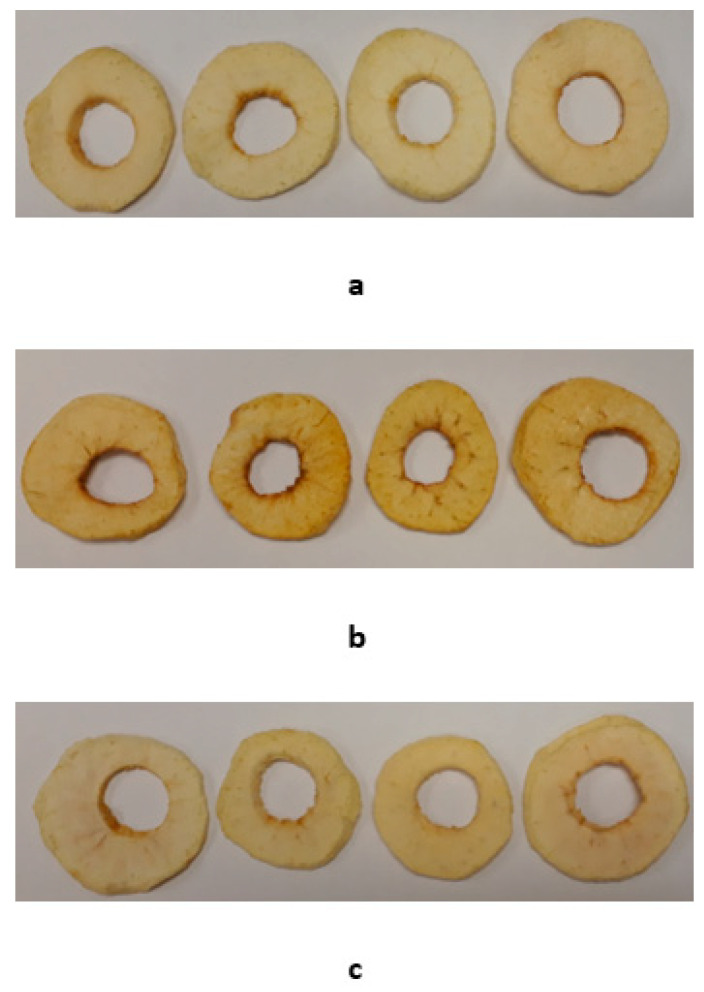
Images of dried apple slices: (**a**) pre-treated with CMO, (**b**) control and (**c**) pre-treated with CMOP at 0 days storage.

The key physical properties of dried fruit are physical appearance and colour, which are the initial assumptions of food quality by consumers [52]. According to [51], these physical properties have become important quality characteristics in consumers perception of the quality of dehydrated apples. The sensory parameter most commonly used to substantiate consumer acceptance of dried fruit is colour [47,51,52]. Therefore, colour becomes one of the main quality aspects in consumer acceptability of dried fruit. The dried sliced apples pre-treated with CMO obtained the highest acceptability score (6.8 = liked moderately) for colour. *Moringa* is an excellent source of polyphenols (flavonoids, phenolic acid and tannins), of which the highest concentration is found in the leaves [53]. Thus, the MOLEP’s antioxidant properties can be attributed to the polyphenols present in the leaves. Citric acid’s ability to lower pH because it can chelate metal (copper) is responsible for minimizing the browning of the dehydrated sliced apples [31,32]. This study showed that the CMO dehydrated sliced apples colour (L*, a* and b*) and whiteness was better than CMOP and the control. Since colour is one of the main aspects of consumers’ perception of quality, it might be the reason why the CMO dried apple slices was liked more by the consumers. Figure 5 illustrates some of the dried apple slices used for the consumer acceptability study.

According to [54], *Moringa oleifera* leaf powder can result in unfavourable consumer acceptability due to its unfamiliar sensory characteristics. The same study indicated that an increase in Moringa oleifera leaf powder resulted in a decrease in acceptability of maize porridge, but at a 1% dosage of *Moringa oleifera* leaf powder, the soft porridge was acceptable by consumers. A study by [39] showed that a 0.1% *Moringa oleifera* leaf extract addition to goat patties was sensorially acceptable. Also, according to [55], citric acid is an organic acid naturally present in fruit and does not impact the flavour of plant products negatively. This consumer acceptability study agrees with these studies since the taste of the dehydrated sliced apples was not significantly (*p* = 1.61) affected by the 2.0% CA and 0.1% MOLEP contained in the pre-treated (CMO and CMOP) water solutions. 

## 4. Conclusions

The combination of 2.0% CA and 0.1% MOLEP (CMO) effectively minimized the redness and yellowness of the dried apple slices over the storage period. Even though the CMOP dried apple slices showed some positive results in minimizing the colour over the storage time, the CMO dried apple slices demonstrated better results. The CMO and CMOP pre-treatments effectively decreased the yeast and mould count of the dehydrated sliced apples. The increase in moisture during the storage period affected the extensibility of the pre-treated dried apple slices negatively. The osmophilic yeasts, moulds and *E. coli* of the CMO and CMOP were within the limits as prescribed by the Codex Alimentarius Commission (2003) at the beginning and end of the storage time and therefore the dried apple slices were safe for consumption. The A_w_ of the dehydrated sliced apples pre-treated with CMO was below the limit conducive to microbial growth and thus is an additional guarantee of food safety for dried sliced apples pre-treated with 2.0% CA and 0.1% MOLEP (CMO). Furthermore, the shelf life of 3 months (120 days) was accomplished. The consumer acceptability test established that colour and taste of the dehydrated sliced apples pre-treated with the 2.0% CA and 0.1% MOLEP (CMO) water solution were liked moderately, and the texture was liked slightly, and therefore acceptable to consumers. Therefore, the objectives to establish storage stability and consumer acceptability of dried sliced apples pre-treated with the combination of the weak acid and *Moringa oleifera* leaf extract powder were accomplished.

## Figures and Tables

**Table 1:** Microbiological test methods information ^1^.

Microorganisms	Method	Plating Technique	Growth Media	Incubation Temperature	Incubation Period
Osmophilic yeast	ISO 21527-2:2008	Spread	DG 18	25 °C	5 days
*Escherichia coli*	ISO 16649-2:2001	Pour	TBX	44 °C	24 h
Yeast and moulds	NFV 08-059:200	Pour	YGC	25 °C	5 days

^1^ DG 18 = dychloran 18% concentration glycero agar; TBX = Tryptone Bile-X glucuronide agar; YGC = yeast extract glucose chloramphenicol agar.

**Table 2:** Impact of pre-treatment on the colour parameters of the dried apple slices ^1^.

	Colour Parameters								
	Lightness (L*)			Redness (a*)			Yellowness (b*)		
	Storage Time (Day)			Storage Time (Day)			Storage Time (Day)		
Pre-Treatment	0	60	120	0	60	120	0	60	120
Control	79.6 ± 3.5 ^a^	77.2 ± 2.6 ^a^	75.0 ± 5.6 ^a^	4.8 ± 2.3 ^a^	4.8 ± 2.0 ^a^	7.0 ± 3.3 ^a^	33.3 ± 4.4 ^a^	34.2 ± 2.7 ^a^	33.8 ± 3.6 ^a^
CMO	85.6 ± 1.5 ^b^	85.1 ± 2.6 ^b^	80.1 ± 2.6 ^b^	1.5 ± 0.7 ^b^	0.9 ± 0.9 ^b^	3.9 ± 2.0 ^b^	17.9 ± 1.4 ^b^	21.7 ± 3.2 ^b^	28.4 ± 4.4 ^b^
CMOP	80.8 ± 2.8 ^a^	80.1 ± 2.9 ^c^	78.4 ± 2.5 ^a^	4.0 ± 1.6 ^a^	3.9 ± 2.0 ^a^	6.2 ± 1.3 ^a^	20.5 ± 2.3 ^b^	21.5 ± 1.9 ^b^	25.1 ± 2.7 ^b^

^1^ Values are mean + standard deviation. Means within a column followed by the same letter are not significantly (*p* > 0.05) different. Control = untreated; CMO = 2.0% CA and 0.1% MOLEP; CMOP = 2.0% CA, 0.1% MOLEP and 0.2% PS.

**Table 3:** Impact of storage time on the colour parameters of dried apple slices ^1^.

	Colour Parameters								
	Lightness (L*)			Redness (a*)			Yellowness (b*)		
	Pre-Treatment			Pre-Treatment			Pre-Treatment		
Storage Time (Days)	Control	CMO	CMOP	Control	CMO	CMOP	Control	CMO	CMOP
0	79.6 ± 3.5 ^a^	85.6 ± 1.5 ^a^	80.8 ± 2.8 ^a^	4.8 ± 2.3 ^a^	1.5 ± 0.7 ^a^	4.0 ± 1.6 ^a^	33.3 ± 4.4 ^a^	17.9 ± 1.4 ^a^	20.5 ± 2.3 ^a^
60	77.2 ± 2.6 ^b^	85.1 ± 2.6 ^a^	80.1 ± 2.9 ^a^	4.8 ± 2.0 ^a^	0.9 ± 0.9 ^a^	3.9 ± 2.0 ^a^	34.2 ± 2.7 ^a^	21.7 ± 3.2 ^b^	21.5 ± 1.9 ^a^
120	75.0 ± 5.6 ^b^	80.1 ± 2.6 ^b^	78.4 ± 2.5 ^a^	7.0 ± 3.3 ^a^	3.9 ± 2.0 ^b^	6.2 ± 1.3 ^b^	33.8 ± 3.6 ^a^	28.4 ± 4.4 ^c^	25.1 ± 2.7 ^b^

^1^ Values are mean + standard deviation. Means within a column followed by the same letter are not significantly (*p* > 0.05) different. Control = untreated; CMO = 2.0% CA and 0.1% MOLEP; CMOP = 2.0% citric acid, 0.1% MOLEP and 0.2% PS.

**Table 4:** Impact of pre-treatment on the colour browning and whitening index of the dried apple slices ^1^.

	Browning Index			Whitening Index		
	Storage Time (Day)			Storage Time (Day)		
Pre-Treatment	0	60	120	0	60	120
Control	57.5 ± 14.0 ^a^	61.0 ± 9.3 ^a^	66.1 ± 18.5 ^a^	60.6 ± 5.6 ^a^	58.6 ± 3.5 ^a^	57.2 ± 6.3 ^a^
CMO	23.9 ± 2.2 ^b^	29.4 ± 6.3 ^b^	46.4 ± 11.0 ^b^	76.9 ± 1.4 ^b^	73.6 ± 3.9 ^b^	65.0 ± 5.0 ^b^
CMOP	32.0 ± 5.0 ^c^	34.0 ± 4.7 ^b^	43.6 ± 7.5 ^b^	71.6 ± 2.9 ^c^	70.4 ± 2.9 ^c^	66.2 ± 3.7 ^b^

^1^ Values are means + standard deviations. Means within a column followed by the same letter are not significantly (*p* > 0.05) different. Control = untreated; CMO = pre-treated with 2% CA and 0.1% MOLEP; CMOP = pre-treated with 2.0% CA, 0.1% MOLEP and 0.2% PS.

**Table 5:** Impact of storage time on the browning and whitening index of the dried apple slices ^1^.

	Browning Index			Whitening Index		
	Pre-Treatment			Pre-Treatment		
Storage Time (Days)	Control	CMO	CMOP	Control	CMO	CMOP
0	57.5 ± 14.0 ^a^	23.9 ± 2.2 ^a^	32.0 ± 5.0 ^a^	60.6 ± 5.6 ^a^	76.9 ± 1.4 ^a^	71.6 ± 2.9 ^a^
60	61.0 ± 9.3 ^a^	29.4 ± 6.3 ^a^	34.0 ± 4.7 ^a^	58.6 ± 3.5 ^a^	73.6 ± 3.9 ^a^	70.4 ± 2.9 ^a^
120	66.1 ± 18.5 ^a^	46.4 ± 11.0 ^b^	43.6 ± 7.5 ^b^	57.2 ± 6.3 ^a^	65.0 ± 5.0 ^b^	66.2 ± 3.7 ^b^

^1^ Values are means + standard deviation. Means within a column followed by the same letter are not significantly (*p* > 0.05) different. Control = untreated; CMO = pre-treated with 2.0% CA and 0.1% MOLEP; CMOP = pre-treated with 2.0% CA, 0.1% MOLEP and 0.2% PS.

**Table 6:** Microbial count of untreated and pre-treated dried apple slices overtime ^#^.

	Osmophilic Yeast (cfu/g)			Yeast (cfu/g)			Moulds (cfu/g)			*E. coli* (cfu/g)		
	Storage Time (Day)			Storage Time (Day)			Storage Time (Day)			Storage Time (Day)		
Pre-Treatment	0	60	120	0	60	120	0	60	120	0	60	120
Control	<100	<100	<100	<1000	130	<10	14,000	<10	<10	<10	<10	<10
CMO	<100	<100	<100	<100	<10	<10	140	<10	<10	<10	<10	<10
CMOP	<100	<100	<100	<10	<10	<10	40	<10	<10	<10	<10	<10

^#^ cfu/g = colony-forming unit per gram. Control = untreated; CMO = pre-treated with 2.0% CA and 0.1% MOLEP; CMOP = pre-treated with 2.0% CA, 0.1% MOLEP and 0.2% PS.

**Table 7:** Effect of the pre-treatment and storage time on the total acidity, A_w_ and moisture of the untreated and pre-treated dried apple slices ^1^.

	Total Acidity			Water Activity (A_W_)			Moisture (%)		
	Storage Time (Day)			Storage Time (Day)			Storage Time (Day)		
Pre-Treatment	0	60	120	0	60	120	0	60	120
Control	2.1 ± 0.2	2.9 ± 0.3	1.5 ± 0.2	0.4 ± 0.0	0.3 ± 0.0	0.4 ± 0.0	7.3 ± 0.7	6.5 ± 0.6	7.8 ± 0.7
CMO	3.8 ± 0.4	4.2 ± 0.5	2.9 ± 0.3	0.3 ± 0.0	0.3 ± 0.0	0.5 ± 0.0	6.0 ± 0.5	6.2 ± 0.6	10.4 ± 0.9
CMOP	3.0 ± 0.3	4.1 ± 0.5	4.1 ± 0.5	0.3 ± 0.0	0.4 ± 0.0	0.4 ± 0.0	6.1 ± 0.5	7.9 ± 0.7	7.7 ± 0.7

^1^ Control = untreated; CMO = pre-treated with 2.0% CA and 0.1% MOLEP; CMOP = pre-treated with 2.0% CA, 0.1% MOLEP and 0.2% PS.

**Table 8:** Impact of treatment on the extensibility of the dried apple slices *.

	Extensibility (mm)		
	Storage Time (day)		
Pre-Treatment	0	60	120
Control	4.7 ± 0.6 ^a^	4.5 ± 0.6 ^a^	6.9 ± 0.6 ^a^
CMO	4.1 ± 0.7 ^a^	5.1 ± 0.4 ^b^	8.0 ± 0.5 ^b^
CMOP	4.7 ± 0.7 ^a^	6.3 ± 0.3 ^c^	7.0 ± 0.5 ^a^

* Values are mean + standard deviation. Means within a column followed by the same letter are not significantly (*p* > 0.05) different. Control = untreated, CMO = pre-treated with 2.0% CA and 0.1% MOLEP, CMOP = pre-treated with 2.0% CA, 0.1% MOLEP and 0.2% PS.

**Table 9:** Impact of storage time on the extensibility of dried apple slices *.

	Extensibility (mm)		
	Pre-Treatment		
Storage Time (Day)	Control	CMO	CMOP
0	4.7 ± 0.6 ^a^	4.1 ± 0.7 ^a^	4.7 ± 0.7 ^a^
60	4.5 ± 0.6 ^a^	5.1 ± 0.4 ^b^	6.3 ± 0.3 ^b^
120	6.9 ± 0.6 ^b^	8.0 ± 0.5 ^c^	7.0 ± 0.5 ^c^

* Values are mean + standard deviation. Means within a column followed by the same letter are not significantly (*p* > 0.05) different. CMO = pre-treated with 2.0% CA and 0.1% MOLEP, CMOP = pre-treated with 2.0% citric acid, 0.1% *Moringa oleifera* leaf extract powder and 0.2% PS.

## Data Availability

All the data is contained in this manuscript.
